# The Peach (*Prunus persica*) *CBL* and *CIPK* Family Genes: Protein Interaction Profiling and Expression Analysis in Response to Various Abiotic Stresses

**DOI:** 10.3390/plants11213001

**Published:** 2022-11-07

**Authors:** Keli Qiu, Haifa Pan, Yu Sheng, Yunyun Wang, Pei Shi, Qingmei Xie, Jinyun Zhang, Hui Zhou

**Affiliations:** 1 Key Laboratory of Genetic Improvement and Ecophysiology of Horticultural Crops, Institute of Horticulture, Anhui Academy of Agricultural Sciences, Hefei 230001, China; 2 School of Life Science, Anhui Agricultural University, Hefei 230036, China

**Keywords:** peach, calcium, abiotic stress, CBL–CIPK pathway

## Abstract

The plant calcineurin B-like protein–CBL interacting protein kinase (CBL–CIPK) signaling pathway is a Ca2+-related signaling pathway that responds strongly to both biological and abiotic environmental stimuli. This study identified eight *CBL* and eighteen *CIPK* genes from peach for the first time. Their basic properties and gene structure were analyzed, and the CBL and CIPK members from Arabidopsis and apple were combined to study their evolutionary relationships. Using RT-qPCR and RNA-seq data, we detected the expression patterns of *PprCBL*s and *PprCIPK*s in different tissues and fruit development stages of peach. Among them, the expression levels of *PprCBL1* and *PprCIPK18* were stable in various tissues and stages. The expression patterns of other members showed specificity between cultivars and developmental stages. By treating shoots with drought and salt stress simulated using PEG6000 and NaCl, it was found that *PprCIPK3*, *PprCIPK6*, *PprCIPK15* and *PprCIPK16* were strongly responsive to salt stress, and *PprCIPK3*, *PprCIPK4*, *PprCIPK10*, *PprCIPK14*, *PprCIPK15*, *PprCIPK16* and *PprCIPK18* were sensitive to drought stress. Three genes, *PprCIPK3*, *PprCIPK15* and *PprCIPK16*, were sensitive to both salt and drought stress. We cloned four *PprCBL* and several *PprCIPK* genes and detected their interaction by yeast two-hybrid assay (Y2H). The results of Y2H show not only the evolutionary conservation of the interaction network of CBL–CIPK but also the specificity among different species. In conclusion, *CBL* and *CIPK* genes are important in peach and play an important role in the response to various abiotic stresses.

## 1. Introduction

Calcium (Ca^2+^) is a ubiquitous second messenger, which is involved in the whole process of plant growth and development and plays a crucial role in plant resistance to stress. In plants, the proteins CBL (calcineurin B-like protein) and CIPK (CBL interacting protein kinase) form important Ca^2+^-decoding complexes to decipher Ca^2+^ signals caused by environmental challenges [1]. The CIPKs are a class of serine/threonine (Ser/Thr) protein kinase. It is an essential family of proteins in Ca^2+^-mediated plant signaling pathways and plays a key role in plant stress response and growth [2]. The response of the CBL–CIPK network to a Ca^2+^ signal can participate in a variety of stresses, such as high salt, osmotic/drought and cold [3]. CIPK initiates a phosphorylation cascade, and its phosphorylation further regulates downstream components to regulate plant growth [2]. For example, the plant Ca^2+^-CBL–CIPK signaling unit maintains the homeostasis of ions/nutrients in root tissue by continuously changing root configuration, expression level and even the activity of mineral nutrient transporters [4]. The signaling network formed by different CBL–CIPK complexes is a key regulatory site in the process of plant signal transduction. Therefore, it has been a hot spot in the field of plant stress research in recent years [5].

CBL proteins have an EF-hand domain, which captures intracellular Ca^2+^. EF-hand is a typical helix-loop-helix motif, containing 12 X•Y•Z•-Y•-X••-Z residues, where the letters represent ligands involved in metal coordination and the dots represents the middle residue [6]. In addition, in recent studies, it was found that the N-terminus of CBLs also contains subcellular localization motifs [7]. All CIPK proteins contain N-terminal and C-terminal domains. The N-terminus is a kinase catalytic domain that contains an activation loop with phosphorylation sites, and the C-terminus is a highly variable regulatory domain that contains NAF/FISL motifs and a phosphatase interaction motif [8]. NAF is a very important domain. When the NAF domain does not bind to CBLs, the N-terminal domain of CIPK itself will be self-inhibited by the FISL domain close to the NAF domain, resulting in no kinase activity [9]. Based on their structural characteristics and evolutionary relationship, CIPK is classified as a sucrose non-fermenting 1 (SNF1)-related kinase and group 3 (SNRK3) [10]. 

Recently, it has been demonstrated in many species that CIPK proteins can resist external stimuli by phosphorylating the N-terminal conserved Ser residues of CBL proteins [11,12], and an increasing number of CBLs and CIPKs enhances plants by regulating various ion concentrations in plant cells of resistance. The CBL–CIPK pathway was first identified in Arabidopsis through the discovery of the SOS pathway, which is composed of *AtCBL4* (*SOS3*), *AtCIPK24* (*SOS2*) and Na^+^/H^+^ antiporter (*SOS1*) [13,14]. AtCBL4 (SOS3) is able to recruit AtCIPK24 (SOS2) to the plasmalemma, where the complex activates the Na^+^/H^+^ antiporter (*SOS1*) and the vacuolar H^+^-ATPase, resulting in enhanced salt tolerance [14]. AtCBL1 and AtCBL9 can activate AtCIPK23 and directly phosphorylate the K^+^ transporter AKT1, thereby enhancing potassium uptake in plants under low potassium conditions [15,16]. In addition, the interaction of CBL2 and CBL3 with CIPK3, 9, 23 and 26 not only directly activates TPK ion channels to improve the adaptability of plants under low potassium conditions [17], but also allows the vacuole to sequester Mg^2+^ in the environment of high magnesium concentration to reduce the toxicity caused by high concentrations of Mg^2+^ and improve the survival of plants [18,19]. In *Brassica napus*, low phosphorus stress strongly induced the expression of *BnCIPK6* in the roots and leaves, and transgenic seedlings overexpressing *BnCIPK6* had more and longer lateral roots under low phosphorus conditions than wild type ones. In addition, overexpression of *BnCBL1* in Arabidopsis enhances plant tolerance to low phosphorus stress. BnCBL1-BnCIPK6 may interact functionally and participate in the response to low phosphorus stress [20]. In wheat, *TaCIPK14* enhanced the activity of the antioxidant system by regulating the expression of stress and defense-related genes, reducing the accumulation of ROS and reducing the damage of cell membranes, thereby enhancing tolerance to cold and salt stresses. In addition, the enhanced salt tolerance of *TaCIPK14*-overexpressing plants was also associated with a decrease in Na^+^ content and an increase in the K^+^/Na^+^ ratio [21]. *CaCIPK3*, a pepper (*Capsicum annuum*) *CIPK* gene, is regulated by CaCBL2 and CaWRKYs through MeJA signal transduction and the antioxidant defense system to enhance drought tolerance [22]. However, the role of peach CBL–CIPK genes in the calcium signaling pathway and its physiological, biochemical and molecular functions in response to environmental stress remains largely elusive. 

In this study, we identified eight *CBL* and 18 *CIPK* genes from peach by database mining and cloning of cDNA sequences of five CBLs and 16 CIPKs. This study not only identified *CBL* and *CIPK* gene family members in the peach genome, but also clarified the interaction of peach CBL and CIPK members. The CBL and CIPK members potentially involved in coercion responses were also accessed by analyzing the expression level changes under the stressed simulation experiments, laying a foundation for future research on the role of the CBL–CIPK pathway in peach stress resistance.

## 2. Results

### 2.1. Characteristics of the Peach CBL and CIPK Gene Families

Hidden Markov model profiles of the EF-hands, NAF and Pkinase domain were used as queries to screen the peach genome (v2.0) to identify potential members of the peach *CBL* and *CIPK* gene family. In total, 8 *CBL* and 18 *CIPK* sequences from the peach genome were found. We also used blastp software v7.0.9.0 to search for candidate CBLs and CIPKs of peach by using Arabidopsis CBLs and CIPK sequences as queries, and the results were identical with those of HMMsearch. SMART and NCBI CDD analysis showed that each peach CBL or CIPK sequence contained EF-hand domains, NAF and Pkinase domains. The eight CBLs and 18 CIPKs were named using the prefix Ppr for P. persica followed by *CBL* and *CIPK* gene family abbreviations and numbered sequentially according to their position on the eight chromosomes. These *CBL* and *CIPK* genes are unevenly distributed on chromosomes, with one *PprCBL* and five *PprCIPK* genes on chromosome 1, two *PprCBL* and *PprCIPK* genes on chromosome 2, one *PprCBL* and *PprCIPK* gene on chromosome 3, three *PprCIPK* genes on chromosome 4, three *PprCBL* genes on chromosome 5, one *PprCBL* and five *PprCIPK* genes on chromosome 6, and two *PprCIPK* genes on chromosome 7 (Table 1). The length of the CBL protein ranges from 212 to 270 amino acids, and that of the CIPK protein ranges from 432 to 490 amino acids. The isoelectric point ranges predicted by CBL and CIPK are 4.70 to 4.93 and 6.50 to 9.15, respectively. The molecular weight ranges of proteins predicted for CBL and CIPK are 24.47 to 30.96 and 47.74 to 55.41 kDa, respectively. Nearly all CIPKs and all CBLs are predicted to be located in the cytoplasm, except for CIPK2, which is predicted to be located in the nucleus.

### 2.2. Phylogenetic, Structure and Motifs Analysis of PprCBL and PprCIPK Family Genes

**Figure 1:**
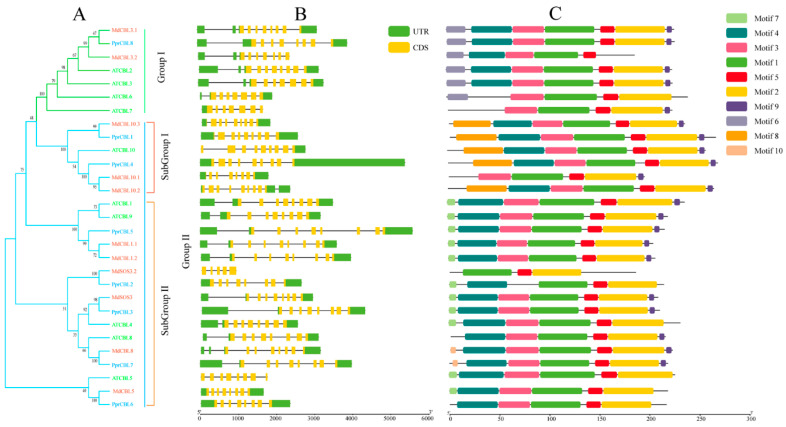
Phylogenetic, structure and motif analysis of CBL gene families from peach, Arabidopsis and apple. (**A**) The phylogenetic analysis of CBL; different colored arcs represent different subgroups. Prefixes ‘Ppr’, ‘Md’ and ‘At’ indicate CBL proteins from Prunus persica, Malus domestica and Arabidopsis, respectively. (**B**) The gene structure analysis of the CBL members; the green rectangle represents the untranslated region (UTR), and the yellow rectangle represents the coding sequence (CDS). (**C**) The conserved motif analysis of each CBL member and different colored rectangles represent different motifs.

**Figure 2:**
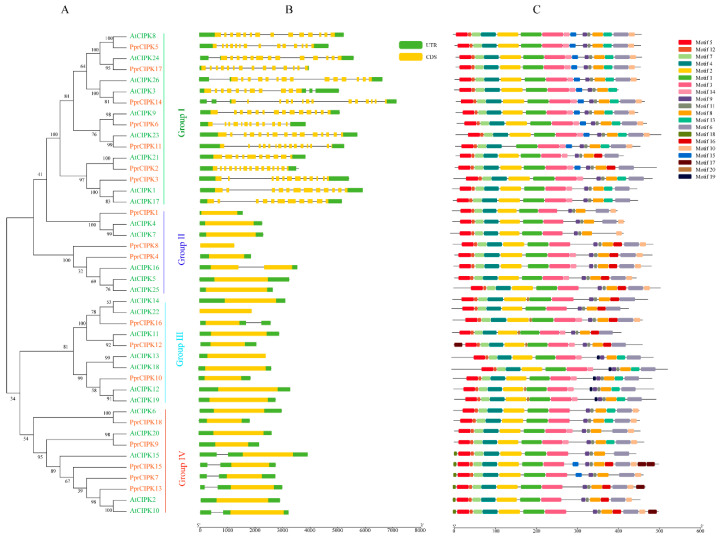
Phylogenetic, structure and motif analysis of CIPK gene families from peach and Arabidopsis. (**A**) The phylogenetic analysis of CIPK; different colored arcs represent different subgroups. (**B**) The gene structure analysis of the CIPK members; the green rectangle represents the untranslated region (UTR), and the yellow rectangle represents the coding sequence (CDS). (**C**) The conserved motif analysis of each CIPK members; different colored rectangles represent different motifs.

To better understand the evolutionary relationships within all the *CBL* and *CIPK* genes in peach and other species, phylogenetic analysis was conducted for these two families (Figure 1 and Figure 2). Ten CBL protein and twenty-six CIPK protein sequences from Arabidopsis and eleven CBL protein sequences in apple were compared using the MEGA X program. 

As shown in Figure 1A, all of the CBL proteins could be divided into two groups: I and II. Similar to Arabidopsis, for peach, group I contained only one member, *PprCBL8*; group II contained the remaining PprCBL members, *PprCBL1/2/3/4/6/7*. The results of CBL gene structure analysis are shown in Figure 1B, where all CBLs shown differ greatly in introns length, but members belonging to the same group have similar intron–exon composition patterns. Most of group 1, subgroup 1 and subgroup 2 members contain a characteristic motif, which are motif 6, motif 8 and motif 7, respectively, reflecting the conservation of these groups in structure. The analysis of CBL protein sequences revealed that motif 1, motif 2, motif 3, motif 4 and motif 5 were present in all sequences, indicating the conservation of these motifs in evolution. The conserved motif sequences are displayed in Table S1. 

The analysis results of CIPK members are shown in Figure 2. The phylogenetic tree analysis was composed of CIPK members of *Arabidopsis* and peach. As shown in Figure 2A, they were divided into three groups according to the phylogenetic tree. Groups I, II, III and IV contain 7, 3, 3 and 5 PprCIPK members, respectively. The analysis of gene structure revealed that the intron–exon structure of CIPK was highly differentiated, which could be divided into intron-poor and intron-rich (group 1) categories (Figure 2B). Among the 11 PprCIPK members in the intron-poor group, 7 members contained no introns and the remaining 4 members contained only one intron in their gene structure. For the intron-rich group (group 1), the intron number of these members ranged from 11 (*PprCIPK3*) to 15 (*PprCIPK14*). The results of conserved motifs (Table S2) analysis shown in Figure 2C indicate that the CIPK members shared relatively conserved motifs, which hardly distinguished each group. 

### 2.3. Expansion Patterns of the Peach CIPK Gene Family

**Figure 3:**
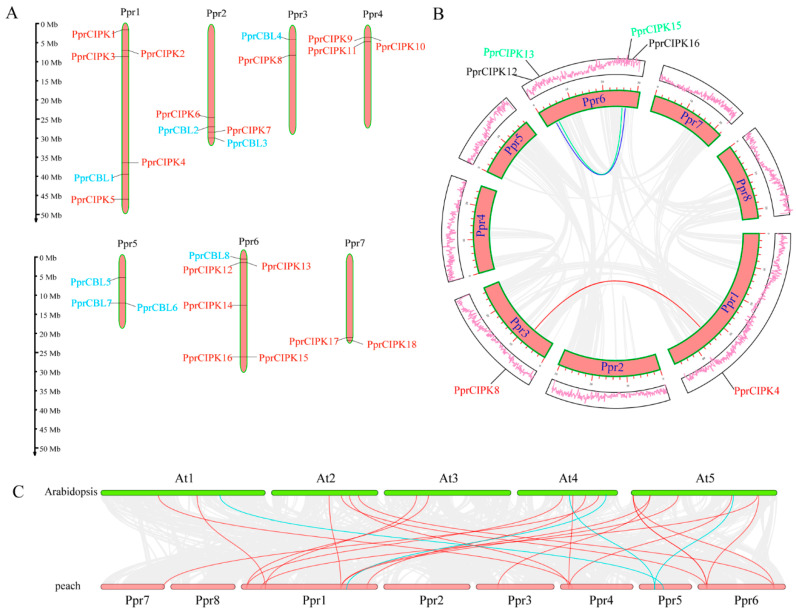
Genomic analysis of peach *CBL* and *CIPK* genes. (**A**) Distribution of CBL and CIPK genes in the peach genome. The orange bars represent peach chromosomes. (**B**) Collinearity analysis of the CBL and CIPK members in the peach genome. The lines represent the segmentally duplicated pair. (**C**) Comparison of collinearity between peach and Arabidopsis genomes. The red lines indicate collinearity between peach and *Arabidopsis* CIPK members, and the blue lines indicate collinearity between peach and *Arabidopsis* CBL members.

Gene families are usually created and maintained by tandem and segmental gene duplication. Tandem duplication and segmented duplication likely play different roles in different gene families [23]. Based on the locations and phylogenetic relationships of *PprCBL* and *PprCIPK* family genes, we found three *CIPK* tandem repeat clusters (*PprCIPK9/10*, *PprCIPK12/13* and *PprCIPK15/16*) located on chromosomes 4 and 6 (Figure 3A). However, no tandem repeat cluster was found for the *PprCBL* family. According to the collinearity analysis results (Figure 3B), three segmentally duplicated pairs, *PprCIPK12/16*, *PprCIPK13/15* and *PprCIPK4/8*, were found for the *PprCIPK* famly but no collinear gene pairs were found for the *PprCBL* family.

Comparison of the CBL and CIPK genes between peach and Arabidopsis can provide insights into the evolutionary history of this gene family. Collinearity analysis between Arabidopsis and peach showed that CBLs and CIPKs were encapsulated within a wide range of collinearity, which is highlighted in Figure 3C. The final collinearity included four pairs of CBLs (blue lines) and twenty pairs of CIPKs (red lines). This finding suggests that these gene pairs are likely generated from the same ancestral gene and may share the same gene function.

### 2.4. Expression Profiles of Peach CBL and CIPK Genes in Different Organs and Fruit Developmental Stages

**Figure 4:**
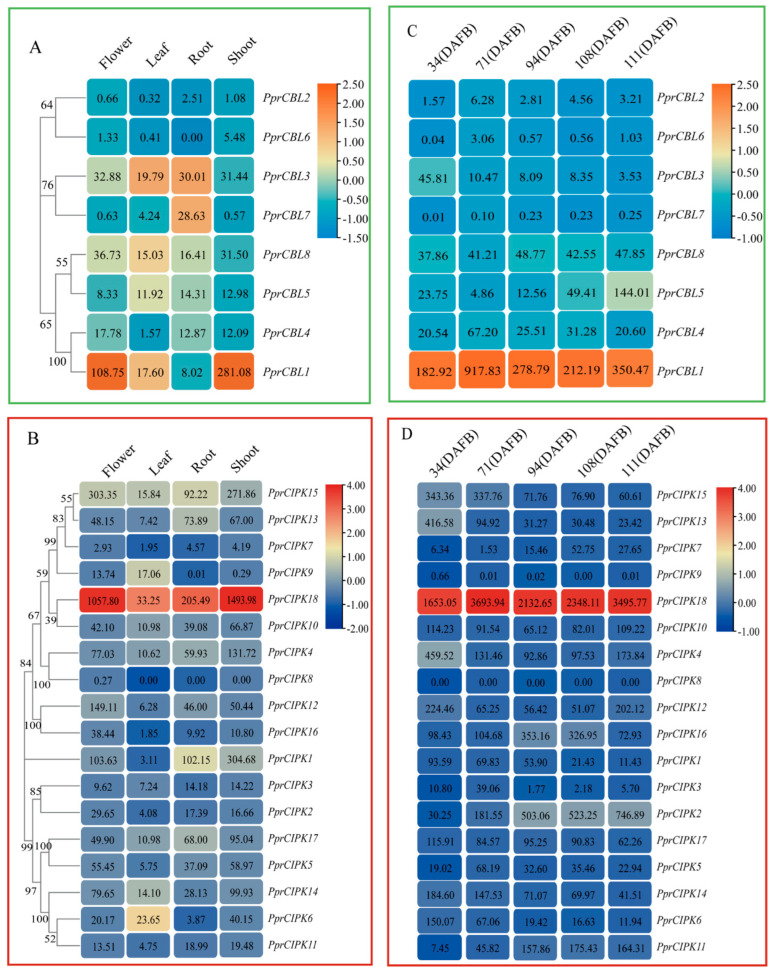
Heatmap analysis of spatiotemporal expression patterns of *CBL* and *CIPK* gene members in peach. (**A**,**B**) CBL and CIPK expression profiles in flowers, leaves, roots and shoots. (**C**,**D**) CBL and CIPK expression patterns in peach at different developmental stages. The value given in the box represents the original gene expression levels (TPM), and the indicator ranges in the upper right corner represent the range of the values after log transformation and normolization.

To further investigate the tissue-specific expression patterns of peach *CBL* and *CIPK* genes, meta-analyses of previously published RNA-Seq data of various tissues and organs, including peach roots, shoots, leaves, flowers and fruits [24,25,26,27], were conducted. A heatmap was constructed according to the gene expression level (TPM) in each tissue. As the map shows (Figure 4), the expression levels of most gene family members showed a tissue-specific pattern. For example, among all tissues tested, *PprCBL7* was only expressed in roots, and the expression levels in other tissues were extremely low; *PprCBL1* was specifically expressed in flowers and shoots; *PprCIPK9* was only expressed in flowers and leaves, and the mRNA level in other tissues was nearly undetectable. In addition to its relatively low expression in leaves, *PprCIPK1* and *PprCIPK18* had an extremely high mRNA abundance in roots, shoots and flowers (Figure 4A,B).

When examining the expression levels of these genes in fruit at different developmental stages, *PprCBL1* and *PprCIPK18* showed constitutively high expression levels at all fruit developmental stages tested (Figure 4C,D). The expression of *PprCIPK2* and *PprCBL5* increased with fruit development and ripening and reached the maximum level at 111 days after full blooming (DAFB). Compared with the other *CBL* and *CIPK* members, the expression levels of these two genes were significantly higher, which suggests that they may play specific roles in fruit development and ripening processes. The expression levels of *PprCBL3*, *PprCIPK6*, *PprCIPK13*, *PprCIPK14* and *PprCIPK15* showed a decreasing trend throughout the whole fruit development process, which indicates that they may play important roles in the early stage of fruit development. During all fruit development stages, the expression levels of *PprCBL6* and *PprCIPK9* were extremely low, while those of *PprCBL7* and *PprCIPK8* were nearly undetectable, which suggests that these genes are not involved in fruit development.

**Figure 5:**
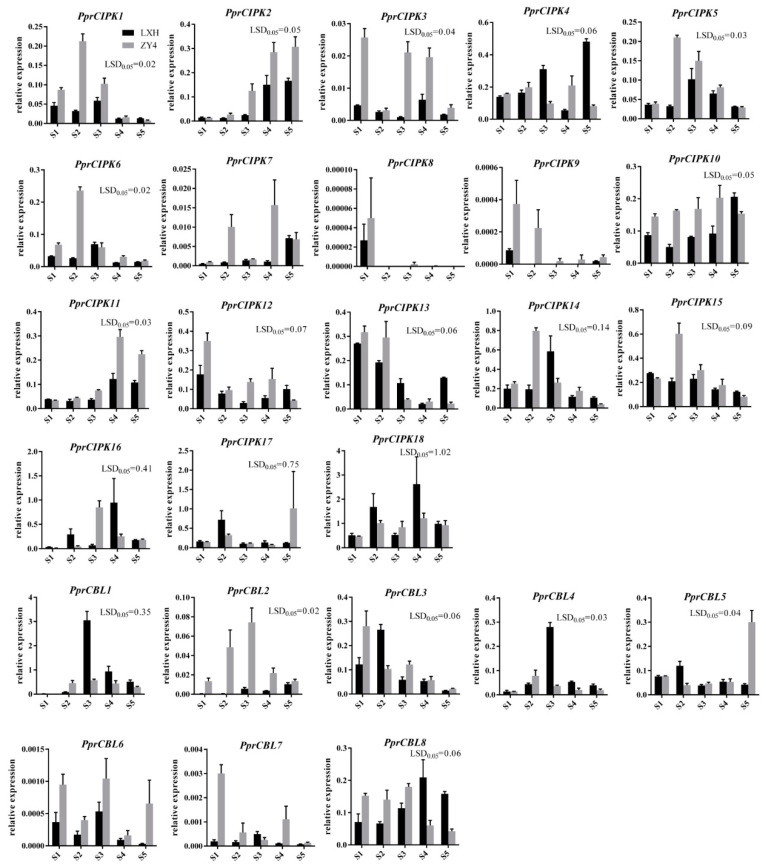
Expression profiles of peach *CBL* and *CIPK* genes during fruit development of “Zhong you 4” and “Li Xia Hong”. The *x*-axis “S1” represents the first exponential growth stage; “S2” represents the pit hardening stage; “S3” represents the second exponential growth stage; “S4” represents the fruit ripening stage; and “S5” represents the full ripening stage. The *y*-axis represents the relative expression of *CBL* and *CIPK* members to the internal reference gene *PprTEF2*. The error bars represent the ± SE of three biological replicates. Any difference between two group means greater than LSD_0.05_ is considered significantly different.

To further determine the expression patterns of *CBL* and *CIPK* genes during fruit development, RT-qPCR was used to detect the expression patterns in “Zhong You 4” and its early ripening bud sport “Li Xia Hong” (Figure 5). The expression pattern of each member was basically consistent with the above RNA-seq results. The mRNA levels of *PprCBL2*, *PprCBL6* and *PprCBL7* were extremely low, and *PprCIPK8* and *PprCIPK9* were undetectable during peach fruit development in the two varieties. The genes with the highest expression in the RNA-seq data, *PprCBL1* and *PprCIPK18*, also showed the highest expression levels in RT-qPCR assays. Interestingly, the expression patterns of some genes were divergent between the two species examined. For example, the expression level of *PprCBL5* was low and stable at the S1-S4 developmental stages of both “Li Xia Hong” and “Zhong you 4” fruits, while it had a significantly higher expression level at the S5 stage of “Zhong you 4” fruit than that of “Li Xia Hong”; the mRNA level of *CIPK4* was similar at the S1, S2 and S4 developmental stages in the two cultivars but showed significant differences at the S3 and S5 stages (Figure 5). These results indicate that the expression levels of *CBL* and *CIPK* genes were affected not only by fruit developmental cues but also by genotypes.

### 2.5. Expression Pattern Analysis of PprCIPK Genes under Different Treatment Conditions

**Figure 6:**
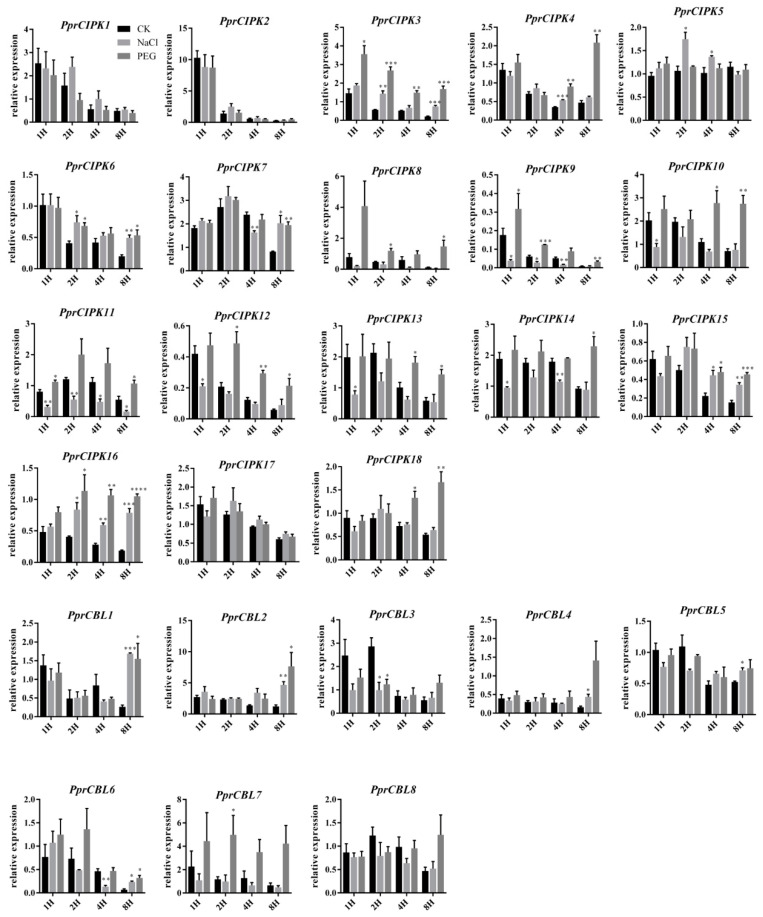
Expression patterns of the peach *CBL* and *CIPK* genes in the leaves of cv. “Li Xia Hong” under salt and drought stresses. The *x*-axis represents the treatment time. The *y*-axis represents the expression of CBL and CIPK members relative to mRNA abundance at 0 h after treatment. The error bars represent the ± SE of three biological replicates. Asterisks denote significant differences between salt or drought stress and the control by Student’s *t* test (*p* ≤ 0.05).

In addition, RT-qPCR was used to analyze the expression pattern of *PprCBL* and *PprCIPK* genes in shoots under different abiotic stresses (Figure 6). The expression levels of some genes decreased with the extension treatment time in both the control and treatment groups. For example, the expression levels of *PprCBL3*, *PprCBL6*, *PprCIPK1* and *PprCIPK2* consistently decreased during both stress treatments, and the expression of *PprCIPK2* decreased nearly 10-fold after 8 h of treatment compared with after 1 h of treatment, indicating that the gene was strongly inhibited at the mRNA level (Figure 6). Interestingly, the expression of some members was stimulated by the stress treatment. For example, the expression of almost all *CIPK* genes was significantly stimulated by 8 h PEG6000 simulated drought treatment, except for *PprCIPK1*, *PprCIPK2*, *PprCIPK5* and *PprCIPK17*. The expression levels of *PprCIPK4* and *PprCIPK18* were higher after 8 h of drought treatment than after 1 h of drought treatment. Interestingly, only the expression of *PprCBL1* and *PprCBL2* in CBL members increased after 8 h of PEG treatment, while the expression of other members decreased. In NaCl-simulated salt stress treatment, the mRNA levels of some members were significantly decreased after salt stress treatment compared with those of the control group, such as *PprCIPK11* and *PprCIPK14*, whose expression seemed to be inhibited under salt stress. However, the expression of some *PprCBL* and *PprCIPK* members showed a response to salt stress. For example, the mRNA levels of *PprCBL1*, *PprCBL2*, *PprCBL6*, *PprCIPK3*, *PprCIPK6*, *PprCIPK7*, *PprCIPK15* and *PprCIPK16* were significantly higher than those in the control group after 8 h of salt treatment, suggesting the possible roles of these genes in response to salt stress. Interestingly, some genes showed responses to both drought and salt treatments, such as *PprCBL1*, *PprCBL2*, *PprCBL6*, *PprCIPK3*, *PprCIPK15* and *PprCIPK16*, whose expression levels were significantly higher than those in the control group after 8 h of treatment, suggesting their dual roles by integrating different stress signals.

### 2.6. Protein Interactions of Peach CBLs and CIPKs

The calcineurin B-like (CBL) protein family represents a unique set of calcium sensors that help decode calcium transients by interacting with the CBL interacting protein kinase (CIPK) family and regulating calcium transients [10]. According to previous studies, CBL and CIPK are involved in plant resistance to abiotic stress in the form of heterodimeric complexes [16,17]. It is very important to study the interaction between CBL and CIPK proteins in peach. The interaction preference of peach CBL proteins with CIPKs was studied using the yeast two-hybrid system. Five *PprCBLs* and sixteen *PprCIPKs* were cloned into pGADT7 and pGBKT7 vectors, respectively. The sequence information of these genes is shown in Table S3. Empty vectors were used as the controls. After hybridization, all combinations of positive diploid transformers CBLs-CIPKs of each grew well on DDO medium but showed different growth states on QDO/A or QDO/A/X medium. 

**Figure 7:**
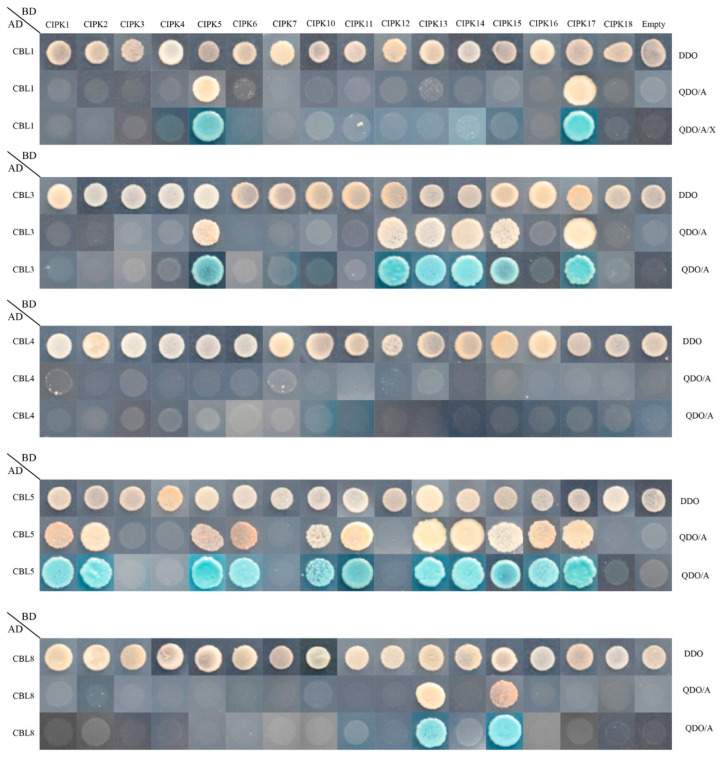
Protein-protein interactions between peach CBL and CIPK proteins. The positive hybrid yeast cells were grown on SD-Trp-Leu (DDO), SD-Trp-Leu-His-Ade + AbA (QDO/A) and SD-Trp-Leu-His-Ade + AbA media supplemented with X-α-Gal (QDO/A/X) to check the interactions.

As shown in Figure 7, in total, 21 pairs of CBL–CIPK interactions were found. Among them, PprCBL1 exhibited strong interactions with PprCIPK5 and PprCIPK17. PprCBL3 interacted with 6 CIPKs, namely PprCIPK5, PprCIPK12, PprCIPK13, PprCIPK14, PprCIPK15 and PprCIPK17. PprCBL8 exhibited interactions with PprCIPK13 and PprCIPK15. It is worth noting that PprCBL5 interacted with the maximum number of PprCIPKs, including PprCIPK1, PprCIPK2, PprCIPK5, PprCIPK6, PprCIPK10, PprCIPK11, PprCIPK13, PprCIPK14, PprCIPK15, PprCIPK16 and PprCIPK17. Interestingly, CBL4 did not interact with any of the 16 CIPKs. These results indicated that, like in Arabidopsis, the CBL–CIPK protein interactions also widely exist in peach. 

To further analyze the relationships of CBL–CIPK pairs identified by Y2H, the co-expression networks of CBLs and CIPKs were calculated using the Pearson correlation coefficient (PCC) according to RT-qPCR results of fruits at different developmental stages (Figure 5) and shoots under different abiotic stresses (Figure 6) (Tables S4 and S5, respectively). Surprisingly, only 38% of the 21 CBL–CIPK pairs identified by Y2H showed positive correlation for the gene expression profiles of fruits at different developmental stages. However, the ratio of positive correlation rose to 81% (19 of 21 pairs) for the mRNA levels in leaves under different abiotic stresses (Table S4), and 14 pairs of them had moderate to strong positive correlations (PCC > 0.4, Table S5). These results indicate that *PprCBLs* and *PprCIPKs* tended to be co-expressed under abiotic stresses, which may facilicate their protein dimerization. Our study provides new proofs that CBL and CIPK participate in abiotic stress reponses by forming a sophisticated network coupling protein interactions and co-expression in mRNA levels, which finally results in orchestrated Ca^2+^ signaling processes.

## 3. Discussion

Calcium plays an important role in the regulation of plant growth, and Ca^2+^ is the second messenger of plants in response to external stimuli [28]. A unique set of calcium sensor proteins, calcineurin b-like (CBL), interacts with CBL-interacting protein kinase (CIPK) to decode the intracellular Ca^2+^ signature. The CBL–CIPK gene family is composed of plant-specific and calcium signaling genes and is involved in indispensable signaling modules in various stress signaling pathways. The physiological functions of some CBL–CIPK pairs have been identified in a variety of plants [29,30,31,32], but less information on CBL–CIPK in peach trees has been reported. Therefore, the peach CIPK-CBL gene family members were identified by genome-wide bioinformatics analysis. In this study, 8 CBL and 18 CIPK members were identified in the peach genome, among which 5 CBL and 16 CIPK genes were successfully cloned, and the interactions between peach CBLs and CIPKs were analyzed using the yeast two-hybrid system. In addition, the expression of each member in the peach fruit development period and the expression patterns under salt and drought stress treatments were also determined.

The ratio of the *CBL* and *CIPK* gene numbers were supposed to be kept with N:N to avoid stoichiometric differences according to the gene balance hypothesis [33]. However, whole-genome and small-scale duplications together with gene lost after duplication have reshaped the pattern of gene numbers of the two gene families. In unicellular green algae, only one gene family member was found for both the *CBL* and *CIPK* gene families [34,35]. As species evolve, the ratio of *CBL* and *CIPK* gene numbers turns into 5:7 and 10:26 in moss and Arabidopsis, respectively [7]. Compared with the *CBL* and *CIPK* gene number ratio (9:17) of *Vitis vinifera*, a species that did not experience recent whole-genome duplication [36], Arabidopsis has a similar number of *CBL* but a much larger number of *CIPK* [7]. Similarly, as in *Vitis vinifera*, peach also did not experience recent whole-genome duplication event after the gamma (γ) whole-genome triplication in core eudicots, and our study showed that peach had a similar gene number ratio (8:18) of *CBL* and *CIPK* to *Vitis vinifera*, indicating the limited effect of segmental and tandem duplication for the two families after the divergence of *Vitaceae* and *Rosaceae*. It was interesting that as in *Vitis vinifera*, peach also has a similar number of *CBL* but a much smaller number of *CIPK* compared with Arabidopsis. It seems that the *CBL* family showed much larger evolutionary constraints on gene duplicate retention than *CIPK*. Another proof of this hypothesis is that after the recent whole-genome duplication event during the evolutionary split of the genera *Malus* and *Prunus*, *Malus* x *domestication* (apple) has 11 *CBL* [37] and 34 *CIPK* [38] gene members, indicating evident gene lost events after gene duplication of *CBL* genes.

Previous studies have shown that CIPK, or the CBL–CIPK complex, plays important roles in responses to environmental stresses [22,30]. For example, AtCIPK24 (SOS2) enables Arabidopsis to resist salt stress by interacting with AtCBL10 (SOS3) [29]. The homologous gene *PprCIPK5* with *AtCIPK24* and *PprCBL1* with *AtCBL10* also showed a response to salt stress, and their expression levels were significantly higher those that of the control group after 4 h of salt stress treatment. AtCIPK23 regulates drought resistance by binding to AtCBL1 and AtCBL9 [39]. *PprCIPK11*, a homolog of *AtCIPK23*, and *PprCBL5*, a homolog of *AtCBL1* and *AtCBL9*, showed stress responsiveness in simulated drought treatment. Moreover, CBL–CIPK complexes are also involved in plant growth or organ development. For example, CBL2/CBL3-CIPK12 complexes play critical roles as regulators of vacuolar dynamics and polarized pollen tube growth [40]. It is interesting that CBL–CIPK complexes also participate in fruit development and ripening. For example, the MdCBL1–MdCIPK13 complex participates in the regulation of sugar accumulation in apple fruits [41]. In grapevine, two CBL–CIPK calcium sensor (CBL) pairs, VvCIPK04–VvCBL01 and VvCIPK03–VvCBL02, may induce fruit veraison (the inception of ripening) by activating the grapevine inward K^+^ channel *VvK1.2* [42]. In peach fruits, *PprCIPK2*, *PprCIPK11* and *PprCBL5* showed up-regulated expression patterns during fruit ripening stages (Figure 4 and Figure 5), which indicated their potential functions in fruit ripening processes. It is worth noting that in peach, coordinated expression of *CBL–CIPK* pairs was found in shoots under salt and drought stresses, but not in normal growing fruits. A coordinated expression pattern of *CBL* and *CIPK* under stress conditions was also reported in *Pisum sativum* in previous reports [43]. One possible explanation for this phenomenon is that *CBL*s and *CIPK*s tend to be co-expressed under abiotic stresses, which may facilitate their protein dimerization.

CBL and CIPK usually form the CBL–CIPK protein complex to participate in Ca^2+^ sensor kinase-related signaling cascades responding to various abiotic and biotic stresses in plants, which represents a basic CBL–CIPK signaling paradigm [1]. Previous studies have found that AtCBL1/AtCBL9 interact with AtCIPK23 to regulate K+ and NO3−[28], and AtCBL1/AtCBL9 can also regulate the ROS signaling pathway by phosphorylating RBOHF by complexing with AtCIPK26 [44]. In peach, PprCBL5, which is homologous to AtCBL1/AtCBL9, was also shown to interact with PprCIPK6 and PprCIPK14, which are homologous to AtCIPK23, and PprCBL5, PprCIPK6 and PprCIPK14 also showed stress responsiveness. PprCBL1 interacts with PprCIPK5, and their respective homologues AtCBL3 and AtCIPK24 also interact [29]. It is worth noting that two CIPK proteins encoded by two duplicated genes, *PprCIPK5* and *PprCIPK24*, were both able to interact with PprCBL1, PprCBL3 and PprCBL5 (Figure 7). *PprCIPK5*/*PprCIPK24* and their orthologs in Arabidopsis *CIPK8*/*CIPK24* belong to a clade named “green algal-type” CIPK including all green algal CIPKs identified with high confidence [45]. This clade showed high sequence conservation (72% and 60% AA pairwise identity of moss and Arabidopsis, respectively) for the anciently duplicated gene, and consequently high conservation of protein interaction patterns. For example, moss CIPK6 and CIPK7 of this clade are both able to interact with CBL1, CBL2 and CBL4 [45]. Similarly, Arabidopsis AtCIPK8 and AtCIPK24 of this clade both can bind with AtCBL1 and AtCBL9 [28]. These results reflect the functional conservation of CBL–CIPK during evolution. Moreover, some new CBL–CIPK complexes that have not yet been reported were discovered. For example, PprCBL3 and PprCIPK5 can interact, but no interaction has been reported for their homologous AtCBL4 and AtCIPK8, respectively. This suggests that CBL–CIPK also has species-specific interactions and plays different functions in different species. 

It has been reported that two retained CBLs or CIPKs after gene duplication probably competed to interact with their counterpart CIPK or CBL [7]. For example, AtCIPK5 and AtCIPK25, encoded by two retained Arabidopsis genes that are both highly expressed in shoots under cold stress, are both able to interact with AtCBL2. However, the strategy of asymmetric expression in roots or under osmotic stress is adopted by the two genes to avoid competition in most circumstances [7]. *PprCIPK4* and *PprCIPK8*, which are orthologs of *AtCIPK5* and *AtCIPK25*, respectively, belong to a collinearity block (Figure 3B), indicating the high conservation of this retained duplication. However, unlike *AtCIPK25*, *PprCIPK8* seemed to be silenced in all the organs, including leaf, flower, shoot, root and fruit (Figure 4B,D). Moreover, the CBLs that PprCIPK4 and PprCIPK8 interact with remained unknown. Would the remaining CBLs with extremely low expression levels be capable to form a dimer with PprCIPK4 or PprCIPK8? This question needs to be addressed by further studies.

## 4. Materials and Methods

### 4.1. Identification of the Peach CBL and CIPK Family

To identify candidate CBL and CIPK family members in the peach genome, peach genome sequences (v2.0) and genome annotation files (v2.1) were downloaded from the GDR database (https://www.rosaceae.org/, accessed on 1 July 2021). In addition, the hidden Markov model (HMM) profile of the EF-hands domain (PF13499) NAF domain (PF03822) and Pkinase domain (PF00069) were downloaded from the Pfam (http://pfam.xfam.org/, accessed on 1 July 2021) database, and then we used the EF-hands domain as queries to search the peach CBL members, and the NAF and Pkinase domains as queries to search the peach CIPK members in the peach genome v2.0 using hmmsearch (HMMER, v3.2.1) software [46]. The e-value threshold for full sequence alignment was set to e-10, and only proteins with e-values lower than the threshold were listed as candidate sequences [37,38]. In addition, we used Blastp analysis to identify CBL–CIPK in the peach genome, using all of the CBL–CIPK sequences in Arabidopsis as query sequences to ensure that all CBL and CIPK in peaches are screened thoroughly.

### 4.2. Plant Materials and Treatment

We collected cv. “Li Xia Hong” and cv. “Zhong You 4” fruit from 10-year-old trees planted in Hefei, Anhui Province. Fruit samples were collected at the following developmental stages: S1 (the first exponential growth), S2 (the pit hardening), S3 (the second exponential growth), S4 (fruit ripening) and S5 (full ripeness). All samples were processed immediately after they were brought back to the laboratory, separated and stored at −75 °C for subsequent use. Each sample had three biological replicates, and each replicate contained at least 10 fruits collected from one tree.

The treatments of the peach shoots were conducted according to a previous report [47] with slight modifications. Young shoots were collected at 10 o’clock in the morning from a “Li Xia Hong” tree. Shoots with similar lengths were selected and placed in distilled water for 1 h before the stress treatments. The shoots were transferred to flasks containing salt (300 mM NaCl) or 10% PEG600 (*w/v*) to simulate salt stress and drought stress, respectively, with ddH_2_O as a control. Each treatment group contained 8–10 independent shoots, and 1–2 leaf samples were collected from each shoot after 0, 1, 2, 4 and 8 h of treatment. Samples were frozen in liquid nitrogen immediately after collection and stored at −80 °C until use.

### 4.3. RNA Extraction and RT-qPCR

Total RNA was extracted using the polysaccharide and polyphenol-enriched total RNAprep Pure Plant Plus Kit (Tiangen, Beijing, China) according to the manufacturer’s instructions. RNA was treated with DNase I and then converted to cDNA with a PrimeScript™ RT Reagent Kit and gDNA Eraser (Takara, Dalian 116000, China). RT-qPCR was conducted using TB GREEN^®^ (Takara Bio, Inc., Kusatsu, Shiga Prefecture, Japan) following the manufacturer’s instructions on an ABI StepOne Plus Real-Time PCR system (Applied Biosystems, Foster City, CA, USA). The amplification program was as follows: 95 °C for 30 s, followed by 40 cycles of 95 °C for 5 s and 60 °C for 30 s. The previously reported translation elongation factor 2 gene *PprTEF2* was used as an internal reference gene [48]. The RT-qPCR primer sequences for each gene are shown in Table S6.

### 4.4. Phylogenetic Tree, Collinearity, and Gene Structure Analysis of CBL and CIPK Gene Family Members

Candidate domains and motifs were verified using SMART (http://smart.embl-heidelberg.de/, accessed on 1 April 2022) databases using default parameters. Totals of 10 Arabidopsis CBL protein and 26 CIPK protein sequences were obtained by TAIR (https://www.arabidopsis.org/, accessed on 1 April 2022). Eleven apple CBL protein members obtained from previous studies [37]. The amino acid sequence of CBL of peach, *Arabidopsis* and apple, and the amino acid sequence of CIPK of peach and *Arabidopsis* were aligned using MUSCLE, and then a phylogenetic tree was constructed by MEGA-X software v10.0.5 using the maximum likelihood method and bootstrap test with 1000 replicates. Collinearity analysis within the peach genome and synteny analysis between the peach genome v2.0 and apple genome v1.0 (https://phytozome-next.jgi.doe.gov/, accessed on 1 July 2021) database were conducted using TBtools software v1.098769 [49]. The locations of genes on chromosomes were displayed using GFF files and TBtools software v1.098769. Schematic diagrams of *PprCBL* and *PprCIPK* genes structures were drawn by Gene Structure Display Server (http://gsds.gao-lab.org/index.php, accessed on 1 April 2022). The conserved motifs of PprCBL and PprCIPK proteins were analyzed using the MEME suite online program (https://meme-suite.org/meme/index.html, accessed on 1 April 2022). 

### 4.5. RNA-Seq Data Analysis and Heatmap Construction

We downloaded peach transcriptome data from the Sequence Read Archive (SRA) (https://www.ncbi.nlm.nih.gov/sra, accessed on 1 May 2019 ) to study the relative expression levels of CBLs and CIPKs in different tissues of peach and different developmental stages of peach fruit. The accession number of the transcriptome data at the peach fruit stage is PRJNA576753, and it includes data from 34 days after full blooming (DAFB), 71 DAFB, 94 DAFB, 108 DAFB and 111 DAFB, corresponding to the five developmental stages of peach S1, S2, S3, S4 and S5, respectively [24]. Transcriptome data for roots and leaves from PRJEB12334 [25], flowers from PRJNA726283 [26] and shoots from PRJNA587386 [27] were also used. Fastp software v0.20.1 was used to perform adapter and low-quality read filtering [50]. Then, clean reads were mapped onto the peach v2.0 genome using HISAT2 version 2.2.1 [51] with default parameters. Quantitative analysis of gene expression levels was performed with the R package Rsubread v2.4.3 [52]. Gene expression abundance was calculated as transcripts per kilobase million (TPM) values for each CBL and CIPK gene. The heatmaps representing gene expression levels were calculated and drawn using TBtools software v1.098769 [49].

### 4.6. Yeast Two-Hybrid Assay

The full-length coding sequences of peach CBLs and CIPKs were amplified using the list of primers in Table S6. The PCR products were purified and homologously recombined with the linearized Y2H vectors pGBKT7 and pGADT7 after restriction enzyme digestion as bait and prey, respectively. Yeast transformation and two-hybrid analysis were conducted according to the manufacturer’s instructions (Clontech, PT4084-1, Mountain View, CA, USA). The bait vector and the prey vector (empty vector as the negative control) were transformed into the yeast strains “Y2Hgold” and “Y187”, respectively, using the Frozen-EZ Yeast Transformation II Kit (Zymo RESEARCH). After mating, the diploid yeast cells were cultured on SD-Trp-Leu (DDO), SD-Trp-Leu-Ade-His + AbA (QDO/A) and SD-Trp-Leu-Ade-His + AbA + X-α-Gal (QDO/A/X) media at 30 °C for 3 d in the dark.

## 5. Conclusions

In conclusion, the CBL and CIPK gene families have received increasing attention in recent years, especially their due to their roles in biotic and abiotic stress. Eight peach CBL members and eighteen CIPK members were screened and identified using bioinformatics methods, and their structure and intraspecific and interspecific evolutionary relationships were analyzed. The expression pattern analysis revealed that three CBL (*PprCBL2*, *PprCBL6* and *PprCBL7*) and two CIPK (*PprCIPK8* and *PprCIPK9*) genes were poorly expressed or undetectable in five different peach tissues. PEG6000 and NaCl were used to simulate drought and salt stress treatment of peach shoots, respectively. The expression patterns of PprCIPK members were detected by RT-qPCR. It was found that *PprCIPK3*, *PprCIPK6*, *PprCIPK15* and *PprCIPK16* were strongly responsive to salt stress, and *PprCIPK3*, *PprCIPK4*, *PprCIPK10*, *PprCIPK14*, *PprCIPK15*, *PprCIPK16* and *PprCIPK18* were sensitive to drought. We cloned four PprCBL and several PprCIPK genes and detected their interaction by Y2H, and the results show 21 interacting gene pairs. Moreover, combing with the gene expression data by RT-qPCR, we found a co-expression tendency for the CBL–CIPK pairs under abiotic stresses. Therefore, this study not only identified the PprCBL–PprCIPK interaction network in peach but also laid a foundation for elucidating the function of PprCBL–PprCIPK.

## Figures and Tables

**Table 1:** Characteristics of *PprCBL* and *PprCIPK* genes and the encoded proteins.

Gene Name	Gene ID	Chr.	Genomic Location (bp)	CDS Length (bp)	Protein Length (aa)	MW (kDa)	pI	Localization
*PprCBL1*	Prupe.1G412900	1	35,996,976–35,999,551	771	256	29.73	4.65	Cytoplasmic
*PprCBL2*	Prupe.2G188700	2	22,874,062–22,876,782	648	215	24.47	4.92	Cytoplasmic
*PprCBL3*	Prupe.2G310300	2	29,368,821–29,373,136	639	212	24.47	4.79	Cytoplasmic
*PprCBL4*	Prupe.3G051100	3	3,597,840–3,603,251	813	270	30.96	4.77	Cytoplasmic
*PprCBL5*	Prupe.5G054400	5	5,813,999–5,819,707	642	213	24.54	4.75	Cytoplasmic
*PprCBL6*	Prupe.5G122100	5	12,135,993–12,138,422	642	213	24.82	4.70	Cytoplasmic
*PprCBL7*	Prupe.5G122200	5	12,139,284–12,143,357	657	218	25.20	4.93	Cytoplasmic
*PprCBL8*	Prupe.6G028500	6	2,222,085–2,226,043	681	226	26.05	4.82	Cytoplasmic
*PprCIPK1*	Prupe.1G026400	1	1,839,105–1,840,662	1305	434	47.74	8.85	Cytoplasmic
*PprCIPK2*	Prupe.1G091700	1	6,909,490–6,913,025	1410	469	52.66	7.53	Nuclear
*PprCIPK3*	Prupe.1G105000	1	8,458,368–8,464,307	1380	459	51.29	6.53	Cytoplasmic
*PprCIPK4*	Prupe.1G392900	1	34,974,805–34,976,767	1362	453	50.88	7.99	Cytoplasmic
*PprCIPK5*	Prupe.1G539600	1	44,093,995–44,099,079	1347	448	50.98	7.23	Cytoplasmic
*PprCIPK6*	Prupe.2G195900	2	23,340,327–23,344,917	1344	447	51.31	8.78	Cytoplasmic
*PprCIPK7*	Prupe.2G261600	2	27,062,570–27,065,556	1326	441	49.87	9.15	Cytoplasmic
*PprCIPK8*	Prupe.3G097900	3	7,505,531–7,506,940	1410	469	53.52	8.64	Cytoplasmic
*PprCIPK9*	Prupe.4G062900	4	3,012,360–3,014,902	1395	464	52.11	8.83	Cytoplasmic
*PprCIPK10*	Prupe.4G063100	4	3,017,616–3,019,702	1461	486	54.49	7.58	Cytoplasmic
*PprCIPK11*	Prupe.4G085000	4	4,165,713–4,171,764	1377	458	51.37	8.99	Cytoplasmic
*PprCIPK12*	Prupe.6G043300	6	3,165,706–3,167,991	1398	465	51.98	8.42	Cytoplasmic
*PprCIPK13*	Prupe.6G043700	6	3,184,043–3,186,693	1395	464	52.91	8.52	Cytoplasmic
*PprCIPK14*	Prupe.6G156900	6	13,963,211–13,970,925	1320	439	50.28	6.85	Cytoplasmic
*PprCIPK15*	Prupe.6G290400	6	26,800,421–26,803,360	1473	490	55.41	8.64	Cytoplasmic
*PprCIPK16*	Prupe.6G290700	6	26,826,847–26,829,608	1317	438	49.61	6.50	Cytoplasmic
*PprCIPK17*	Prupe.7G244500	7	20,886,040–20,890,002	1341	446	50.49	8.50	Cytoplasmic
*PprCIPK18*	Prupe.7G261300	7	21,709,634–21,711,533	1299	432	48.61	8.96	Cytoplasmic

## Data Availability

All of the accession numbers of the peach *TIFY* genes in this study can be found in Table 1.

## Supplementary Materials

The following supporting information can be downloaded at: https://www.mdpi.com/article/10.3390/plants11213001/s1, Table S1: The sequences information of CBL each conserved motif; Table S2: The sequences information of CIPK each conserved motif; Table S3: Comparison of CBL and CIPK CDS sequences in reference genomes and LXH; Table S4: Co-expression analysis of CBLs and CIPKs using the Pearson correlation coefficient (PCC) according to RT-qPCR results of fruits at different developmental stages; Table S5: Co-expression analysis of CBLs and CIPKs using the Pearson correlation coefficient (PCC) according to RT-qPCR results of shoots under different abiotic stresses; Table S6: Sequences of primers used for cloning genes and vectors construction and RT-qPCR; Figure S1. Comparison of PprCIPK7 CDS sequences in reference genomes and LXH; Figure S2. Comparison of PprCIPK15 CDS sequences in reference genomes and LXH; Figure S3. Comparison of PprCBL4 CDS sequences in reference genomes and LXH; Figure S4. Comparison of PprCBL5 CDS sequences in reference genomes and LXH.
