# Supplementary material for: The Peach (Prunus persica) CBL and CIPK Family Genes: Protein Interaction Profiling and Expression Analysis in Response to Various Abiotic Stresses
Source: Plants (Basel). 2022 Nov 7;11(21):3001. doi: 10.3390/plants11213001 (PMC9653928; doi:10.3390/plants11213001)
Supplement: Supplementary file 1 [file plants-11-03001-s001.zip › plants-1942756-supplementary.pdf]

Table S1. The sequences information of CBL each conserved motif.

**Motif 1**	**NGVIEFEEFVRALSVFHPNAPJEDKIDFAFRLYDLRQTGFIERZEVKQMV**
Motif 2	DKTFEEADTKKDGKIDKEEWKEFVLKHPSLLKNMTLPYLKDITTTFPSFV
Motif 3	IHKEEFQLALFKTRKKENLFADRVFDLFDVKR
Motif 4	QSPGYEDPEVLASETPFTVNEVEALYELFKKJSSSVIDDGL
Motif 5	ESDMKLSDDVIESIV
Motif 6	MLQCIDGLKHLCASLLRCCD
Motif 7	MGCFSSKA
Motif 8	RSSSLSIGEKJCAVFIPFIAJVEALVFSVAGCFDRRRQEP
Motif 9	NSEVED
Motif 10	FLHCFC

Table S2. The sequences information of CIPK each conserved motif.

**Motif 1**	**DGLLHTTCGTPAYVAPEVJNKKGYDGAKADIWSCGVILFVLLAGYLPFDD**
Motif 2	EDVARKYFQQLISAVDYCHSRGVYHRDLKPENLLLDENGNLKVSDFGLSA
Motif 3	NLMAMYRKIYKAEFKCPPWFSPEAKRLJSRJLDPNPETRITIAEIMEDPW
Motif 4	PNIVRLYEVMATKTKIYFVMEYVKGGELFBKIAKGG
Motif 5	KYELGRLLGZGTFAKVYHARNIETGESVA
Motif 6	GQLSVAAEIFEVTPSLVVVEVKKSGGDTLEYHKFYK
Motif 7	JKVGMIEQIKREISIMRLVRH
Motif 8	DESKRETRFTSKKPASEIISKJEEIA
Motif 9	LNAFDJISLSS
Motif 10	DLRPALKDIVWKWQG
Motif 11	GFDLSGLF
Motif 12	IKVIDKEK
Motif 13	KPMGFKVRKKBYKLRLEGEKE
Motif 14	FKKGYKPIKFYEEDD
Motif 15	NLDDVDAVFDDSEES
Motif 16	KELNLKVKKKKDFKVKLEGKK
Motif 17	QEQZQQQQPPQQEQPEQEEP
Motif 18	MENKGSVL
Motif 19	GSMPRPAS
Motif 20	TDQIRP

Table S3. Comparison of CBL and CIPK CDS sequences in reference genomes and LXH.

**Sequence**	**SNPs or Gaps**
PprCIPK1	0
PprCIPK2	0
PprCIPK3	0
PprCIPK4	0
PprCIPK5	0
PprCIPK6	0
PprCIPK7	1 SNP, “C” to “T” (Fig S1)
PprCIPK10	0
PprCIPK11	0
PprCIPK12	0
PprCIPK13	0
PprCIPK14	0
PprCIPK15	1 SNP, “G” to “A” (Fig S2)
PprCIPK16	0
PprCIPK17	0
PprCIPK18	0

PprCBL1	0
PprCBL3	0
PprCBL4	1 Gap, lost “AAGTATCTTGGT” (Fig S3)
PprCBL5	2 SNP, “A” to “G”; “G” to “A” (Fig S4)
PprCBL8	0

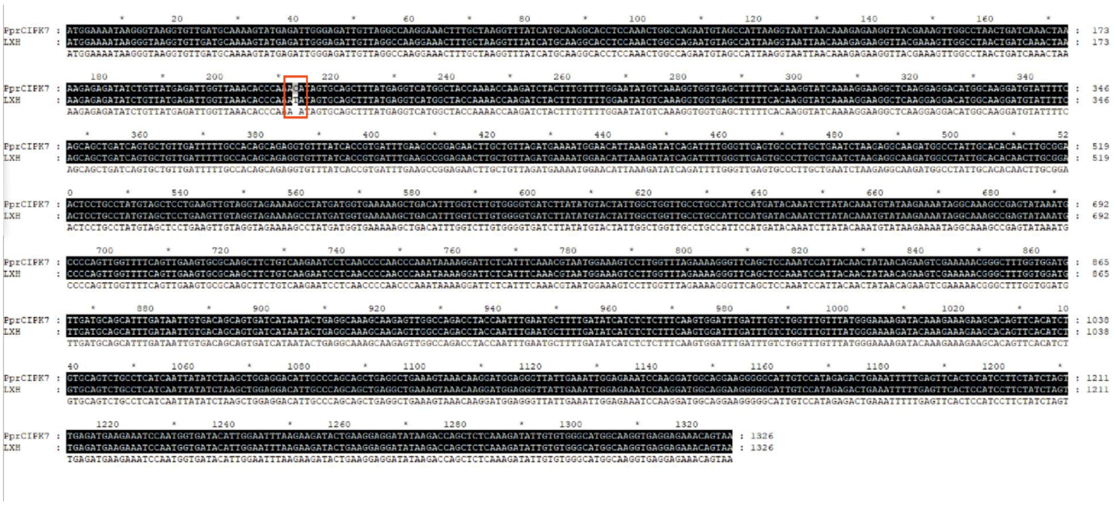

Figure S1. Comparison of *PprCIPK7* CDS sequences in reference genomes and LXH.

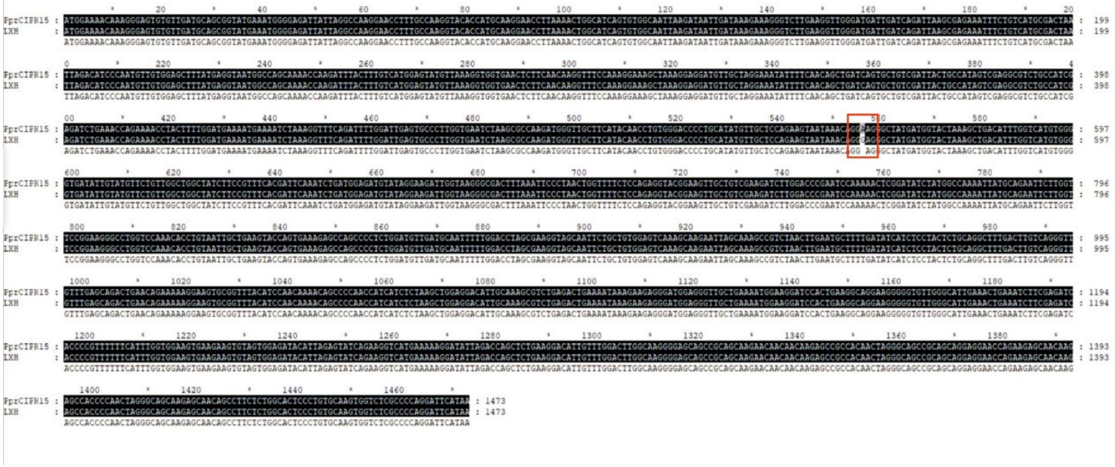

Figure S2. Comparison of *PprCIPK15* CDS sequences in reference genomes and LXH.

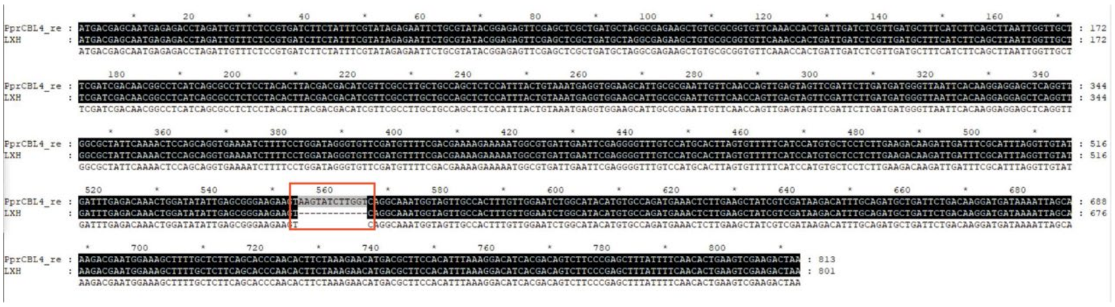

Figure S3. Comparison of *PprCBL4* CDS sequences in reference genomes and LXH.

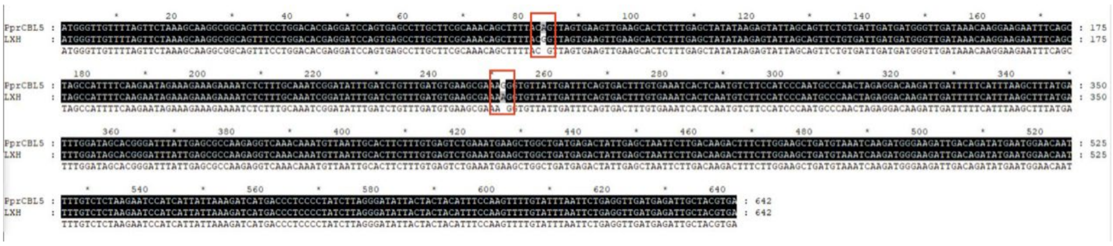

Figure S4. Comparison of *PprCBL5* CDS sequences in reference genomes and LXH.

Table S6. Sequences of primers used for cloning genes and vectors construction and RT-qPCR.

Primer name	Sequence	Description
PpCBL1F	CCGTTTATAGCAATCATAGAGGTGT	primers used for RT-qPCR of PpCBL1
PpCBL1R	CCTCCAGCTCATTAACGGTAAAT	
PpCBL2F	CCAAAACCCCCTTTTCTGTG	primers used for RT-qPCR of PpCBL2
PpCBL2R	AAAAGTTCAAGCTGGAGCTCTTC	
PpCBL3F	GCTGCTGAAACACCCTTTACAGT	primers used for RT-qPCR of PpCBL3
PpCBL3R	TTGTTTTTGAAGAGTGCAAGCTG	
PpCBL4F	CAGCTTAATTGGTTGCTTCGAT	primers used for RT-qPCR of PpCBL4
PpCBL4R	GAACAATTCGCGCAATGCT	
PpCBL5F	GTTCTGTGATTGATGATGGGTTG	primers used for RT-qPCR of PpCBL5
PpCBL5R	TGAGTGATTTCACAAAGTCACTGAA	
PpCBL6F	CTCGAAACATGACGGGGTAATAG	primers used for RT-qPCR of PpCBL6
PpCBL6R	TCACCTCTTCACGTTCGATGAA	
PpCBL7F	CTGTTTAAGAAACTAAGCAGTTCCAT	primers used for RT-qPCR of PpCBL7
PpCBL7R	TACGCTTGATGTCAAACAAGTCA	
PpCBL8F	GAGACAGTGTTCAGCGTGAGTGA	primers used for RT-qPCR of PpCBL8
PpCBL8R	TTGTTTGTCTTGAATAATGCCAGTT	
PpCIPK1F	GAAGGAGGTTGCGCTGAGTTT	primers used for RT-qPCR of PpCIPK1
PpCIPK1R	CAGCTCTCAACTCCTCCCAAAG	
PpCIPK2F	GAGGTGATTGGTACAAAGACCAAA	primers used for RT-qPCR of PpCIPK2
PpCIPK2R	AAAAAGCTTTCTTGCATCTCCTTC	
PpCIPK3F	CCAACGTTGTCAGATTACACGAG	primers used for RT-qPCR of PpCIPK3
PpCIPK3R	AACTGTTGGAAGAGCTTCCTGC	
PpCIPK4F	GCTCAGCCACGGCGATTA	primers used for RT-qPCR of PpCIPK4
PpCIPK4R	CAGCCCTTTCCTTCCCTCA	
PpCIPK5F	AGGCGATACTTCCACCAGCTAA	primers used for RT-qPCR of PpCIPK5
PpCIPK5R	GGCAATGCACTCAGACCAAAA	
PpCIPK6F	ACAGCAAGTGCGGGAAGATG	primers used for RT-qPCR of PpCIPK6
PpCIPK6R	AGAGAATGACCCCACAAGACCA	
PpCIPK7F	GACATTGCCCAGCAGCTGA	primers used for RT-qPCR of PpCIPK7
PpCIPK7R	CTCTATGGACAATGCCCCCTT	
PpCIPK8F	CTCCACCATTTTTCAATGCTTTC	primers used for RT-qPCR of PpCIPK8
PpCIPK8R	AGCACTTGGACGTGAACATCG	
PpCIPK9F	AAGATGGGACCCTGAAATTGC	primers used for RT-qPCR of PpCIPK9
PpCIPK9R	GAGAGGGCTTTAAGTCATGGTCA	
PpCIPK10F	TTTCAGTGAGGAAGAAGGATTGC	primers used for RT-qPCR of PpCIPK10
PpCIPK10R	AACCTCCAAAACCACCAAAGAC	
PpCIPK11F	GTGCACTACCTCAGCAAGTTCG	primers used for RT-qPCR of PpCIPK11
PpCIPK11R	TTTTGCTCCATCATACCCTTTG	
PpCIPK12F	AGGGTGAGGAGGAAGAAGGAGT	primers used for RT-qPCR of PpCIPK12

PpCIPK12R	TCTTCCACATCTCCCTTGAAGG	
PpCIPK13F	AATGGGGATTTTGTCCATAGATG	primers used for RT-qPCR of PpCIPK13
PpCIPK13R	GCCAAACCCAGACTATATCTTGC	
PpCIPK14F	CCAAATTATGTTGCTCCTGAGGT	primers used for RT-qPCR of PpCIPK14
PpCIPK14R	GGTCATAAGATTAGAATCATCAAAAGG	
PpCIPK15F	GACTTGGCAAGGGGAGCAG	primers used for RT-qPCR of PpCIPK15
PpCIPK15R	TTGCTCTTGCTGCCCTAGTTG	
PpCIPK16F	ATGGTAATTTCGTGGTGATGATTG	primers used for RT-qPCR of PpCIPK16
PpCIPK16R	TCGCTGGCTACATTCACTTTGT	
PpCIPK17F	ATGGCTGGATATCTCCCATTTG	primers used for RT-qPCR of PpCIPK17
PpCIPK17R	TGTATCAGAGAATTTGCCTCAGGA	
PpCIPK18F	GGTAGCCAAGGCTGGCAAG	primers used for RT-qPCR of PpCIPK18
PpCIPK18R	CACAAACGACGGCGTCATC	
PpCBL1-ADF	GCCATGGAGGCCAGTGAATTCATGGATTTCTCGGTAAAAAGATCG	primers used for inserting PpCBL1 into vector pGADT7
PpCBL1-ADR	ATTCATCTGCAGCTCGAGCTCTCAGTAGGAAAAGACTTCAAATTCTGA	
PpCBL3-ADF	GCCATGGAGGCCAGTGAATTCATGGGCTGCTATTGGTCAAAG	primers used for inserting PpCBL3 into vector pGADT7
PpCBL3-ADR	ATTCATCTGCAGCTCGAGCTCTCATATTTCTGAATCTTCAACTTCAGAG	
PpCBL5-ADF	GCCATGGAGGCCAGTGAATTCATGGGTTGTTTTAGTTCTAAAGCAA	primers used for inserting PpCBL5 into vector pGADT7
PpCBL5-ADR	ATTCATCTGCAGCTCGAGCTCTCACGTAGCAATCTCATCAACCT	
PpCBL8-ADF	GCCATGGAGGCCAGTGAATTCATGTTGCAGTGCATAGAGGGG	primers used for inserting PpCBL8 into vector pGADT7
PpCBL8-ADR	ATTCATCTGCAGCTCGAGCTCTCAGGTGTCGTCCACTTGTGAG	
PpCIPK1-BDF	ATGGCCATGGAGGCCGAATTCATGGACCAAGGGGTCCCA	primers used for inserting PpCIPK1 into vector pGBKT7
PpCIPK1-BDR	CCGCTGCAGGTCGACGGATCCTCAAACGACGACGTTATGCC	
PpCIPK2-BDF	ATGGCCATGGAGGCCGAATTCATGGGACTTGCCAGCAACA	primers used for inserting PpCIPK2 into vector pGBKT7
PpCIPK2-BDR	CCGCTGCAGGTCGACGGATCCTCAAGACGATGAATAGCCACG	
PpCIPK3-BDF	ATGGCCATGGAGGCCGAATTCATGGTGATCGTAAGAAAAGGTGC	primers used for inserting PpCIPK3 into vector pGBKT7
PpCIPK3-BDR	CCGCTGCAGGTCGACGGATCCTCACACTTGGCTAGTCAACAACC	
PpCIPK4-BDF	ATGGCCATGGAGGCCGAATTCATGGAAGAAAGGACTGTCTTGTTT	primers used for inserting PpCIPK4 into vector pGBKT7
PpCIPK4-BDR	CCGCTGCAGGTCGACGGATCCTCATGAGTCTTGTTGATCACCCT	
PpCIPK5-BDF	ATGGCCATGGAGGCCGAATTCATGGTGGTGAGGAAAGTGGGTA	primers used for inserting PpCIPK5 into vector pGBKT7
PpCIPK5-BDR	CCGCTGCAGGTCGACGGATCCTCAACGCTTTTTACTTTTACTCTTGC	
PpCIPK6-BDF	ATGGCCATGGAGGCCGAATTCATGGAGAAGATGAGTGCGGC	primers used for inserting PpCIPK6 into vector pGBKT7
PpCIPK6-BDR	CCGCTGCAGGTCGACGGATCCTTATTTCCTTGATCCTTCAGTAGTTTC	
PpCIPK7-BDF	ATGGCCATGGAGGCCGAATTCATGGAAAATAAGGGTAAGGTGTTGA	primers used for inserting PpCIPK7 into vector pGBKT7
PpCIPK7-BDR	CCGCTGCAGGTCGACGGATCCTTACTGTTTCTCCTCACCTTGCC	
PpCIPK10-BDF	ATGGCCATGGAGGCCGAATTCATGGCAAACCTCACCACCA	primers used for inserting PpCIPK10 into vector pGBKT7

PpCIPK10-BDR	CCGCTGCAGGTCGACGGATCCCTATTCAGAATCTGAAGGTAGATATGAAG	
PpCIPK11-BDF	ATGGCCATGGAGGCCGAATTCATGTCGTCGAGGTCGGCG	primers used for inserting PpCIPK11 into vector pGBKT7
PpCIPK11-BDR	CCGCTGCAGGTCGACGGATCCTCAAGATGGCAGAGCACCG	
PpCIPK12-BDF	ATGGCCATGGAGGCCGAATTCATGCCAGAGATCGAACAGCAG	primers used for inserting PpCIPK12 into vector pGBKT7
PpCIPK12-BDR	CCGCTGCAGGTCGACGGATCCTCAATCAGCTGGGCAGTGC	
PpCIPK13-BDF	ATGGCCATGGAGGCCGAATTCATGGAGAATAAATCGAATGTGCTG	primers used for inserting PpCIPK13 into vector pGBKT7
PpCIPK13-BDR	CCGCTGCAGGTCGACGGATCCCTATTGTGGCTGATGCTGTTCTAGT	
PpCIPK14-BDF	ATGGCCATGGAGGCCGAATTCATGAATCAACCAAAAATCAAGCG	primers used for inserting PpCIPK14 into vector pGBKT7
PpCIPK14-BDR	CCGCTGCAGGTCGACGGATCCTTACTTCACTTTTTGCATATCCTCCT	
PpCIPK15-BDF	ATGGCCATGGAGGCCGAATTCATGGAAAACAAAGGGAGTGTGTT	primers used for inserting PpCIPK15 into vector pGBKT7
PpCIPK15-BDR	CCGCTGCAGGTCGACGGATCCTTATGAATCCTGGGGCGAGA	
PpCIPK16-BDF	ATGGCCATGGAGGCCGAATTCATGCCGGAGATCGAGGTAGT	primers used for inserting PpCIPK16 into vector pGBKT7
PpCIPK16-BDR	CCGCTGCAGGTCGACGGATCCTTAATTACCGGCGACGTCC	
PpCIPK17-BDF	ATGGCCATGGAGGCCGAATTCATGAAGAAGGTGTCGAGAAAGGTAG	primers used for inserting PpCIPK17 into vector pGBKT7
PpCIPK17-BDR	CCGCTGCAGGTCGACGGATCCTCAGCAAGTCATTGTTCGAAGC	
PpCIPK18-BDF	ATGGCCATGGAGGCCGAATTCATGGCGGATGAAAAAGGCA	primers used for inserting PpCIPK18 into vector pGBKT7
PpCIPK18-BDR	CCGCTGCAGGTCGACGGATCCTCAAGCAGCCGTCGAACTGT	
